# An apparently new virus (family Paramyxoviridae) infectious for pigs, humans, and fruit bats.

**DOI:** 10.3201/eid0402.980214

**Published:** 1998

**Authors:** A W Philbey, P D Kirkland, A D Ross, R J Davis, A B Gleeson, R J Love, P W Daniels, A R Gould, A D Hyatt

**Affiliations:** Elizabeth Macarthur Agricultural Institute, Camden, New South Wales, Australia. Adrian.Philbey@agric.nsw.gov.au

## Abstract

We isolated an apparently new virus in the family Paramyxoviridae from stillborn piglets with deformities at a piggery in New South Wales, Australia. In 1997, the pregnancy rate and litter size at the piggery decreased markedly, while the proportion of mummified fetuses increased. We found serologic evidence of infection in pigs at the affected piggery and two associated piggeries, in humans exposed to infected pigs, and in fruit bats. Menangle virus is proposed as a common name for this agent, should further studies confirm that it is a newly recognized virus.


### An Apparently New Virus (Family *Paramyxoviridae*) Infectious for Pigs, Humans, and Fruit Bats

**Adrian W. Philbey,* Peter D. Kirkland,* Anthony D. Ross,* Rodney J. Davis,* Anne B. Gleeson,* Robert J. Love,† Peter W. Daniels,‡ Allan R. Gould,‡ and Alex D. Hyatt‡**

*Elizabeth Macarthur Agricultural Institute, Camden, New South Wales, Australia; †University of Sydney, Camden, New South Wales, Australia; and ‡CSIRO Australian Animal Health Laboratory, Geelong, Victoria, Australia

We isolated an apparently new virus in the family *Paramyxoviridae* from stillborn piglets with deformities at a piggery in New South Wales, Australia. In 1997, the pregnancy rate and litter size at the piggery decreased markedly, while the proportion of mummified fetuses increased. We found serologic evidence of infection in pigs at the affected piggery and two associated piggeries, in humans exposed to infected pigs, and in fruit bats. Menangle virus is proposed as a common name for this agent, should further studies confirm that it is a newly recognized virus.

Viruses in the family *Paramyxoviridae* have been associated with new diseases in a variety of species, including humans, throughout the world. Morbilliviruses related to canine distemper virus have caused disease outbreaks in seals, dolphins, porpoises, and lions (1-6). Equine morbillivirus (EMV) (also called Hendra virus or bat paramyxovirus), responsible for the deaths of horses and humans with respiratory or neurologic disease in Australia (7,8), is thought to have originated from fruit bats (*Pteropus* sp.) (9-11). La Piedad Michoacan paramyxovirus (blue eye paramyxovirus) causes encephalitis and death in piglets and reproductive disease in adult pigs in Mexico (12). We have isolated an apparently new virus in the family *Paramyxoviridae* from stillborn piglets with abnormalities of the brain, spinal cord, and skeleton at a commercial piggery with 2,600 sows in New South Wales, Australia. Serologic studies indicate that at least two humans exposed to affected pigs have been infected with the virus, possibly with resultant illness, and that fruit bats are a potential source of infection.

From mid-April to early September 1997 at the affected piggery, the farrowing rate decreased from an expected 82% to 60%; the number of live piglets declined in 27% of litters born; the proportion of mummified and stillborn piglets, some with deformities, increased; and occasional abortions occurred. The disease occurred sequentially in all four breeding units at the piggery, affecting the progeny of sows of all parities and gilts. No disease was observed in postnatal animals of any age. Affected stillborn piglets frequently had severe degeneration of the brain and spinal cord (which were almost absent in some piglets), arthrogryposis, brachygnathia, and occasionally fibrinous body cavity effusions and pulmonary hypoplasia. Histologic examination of the brain and spinal cord showed extensive degeneration and necrosis of gray and white matter associated with infiltrations of macrophages and other inflammatory cells. Neurones contained intranuclear and intracytoplasmic inclusion bodies. Some piglets had nonsuppurative myocarditis.

A virus producing cytopathic effects in cell cultures, including vacuolation of cells and formation of syncytia, was isolated at the Elizabeth Macarthur Agricultural Institute, Menangle, in baby hamster kidney cells (BHK21) from lung (n = 9 isolates), brain (n = 8), and heart (n = 5) of affected piglets. The isolates have been shown by electron microscopy to be morphologically consistent with viruses in the family

Address for correspondence: Adrian W. Philbey, Elizabeth Macarthur Agricultural Institute, PMB 8, Camden, New South Wales 2570, Australia; fax: 61-2-46-40-6429; e-mail: Adrian.Philbey@agric.nsw.gov.au.

*Paramyxoviridae*, which comprise spherical to pleomorphic particles 30 nm to more than 100 nm long, contain "herringbone" nucleocapsids with a diameter of 19 ± 4 nm and a pitch of 5.8 ± 0.4 nm, and are surrounded by an envelope with a single fringe of surface projections 17 ± 4 nm long (Figure). The virus grows in a wide range of cell types from many species, including porcine and human cells, and is nonhemadsorbing and nonhemagglutinating, using erythrocytes from several species; in this respect, the virus differs from La Piedad Michoacan paramyxovirus, which is hemagglutinating (13). Studies at the Australian Animal Health Laboratory, Geelong, indicate that the virus is probably a new member of the family *Paramyxoviridae.* EMV and La Piedad Michoacan paramyxovirus have been excluded on the basis of absence of specific polymerase chain reaction products and limited sequencing, and EMV has been further excluded on the basis of lack of serologic cross-reactivity and differing appearance by electron microscopy. Serologic tests at the Elizabeth Macarthur Agricultural Institute and the Australian Animal Health Laboratory on sera from sows have also excluded other porcine reproductive pathogens, such as ruminant and porcine pestiviruses (including classic swine fever virus), porcine reproductive and respiratory syndrome (Lelystad) virus, porcine parvovirus, encephalomyocarditis virus, and Aujeszky's disease virus. Pestivirus infection was also excluded by negative antigen capture enzyme-linked immunosorbent assay results on tissues from affected piglets.

A high proportion (>90%) of serum collected from pigs of all age categories at the affected piggery (n = 88) from May to September 1997 contained high titers (≥ 256) of neutralizing antibodies against the virus. In contrast, serum and plasma samples collected from pigs at the affected piggery before May 1997 (n = 120) tested negative. Porcine sera (n = 50) from two piggeries that receive only weaned pigs from the affected piggery neutralized the virus at dilutions of 16 to 4,096. Virus-neutralizing antibodies were also detected in body cavity fluids from some stillborn piglets. Testing of porcine sera (n = 1,114) from other piggeries throughout Australia, including piggeries with reproductive problems, indicates that infection is confined to the affected piggery and the two associated piggeries.

Serum from two humans—one working at the affected piggery and one working at one of the associated piggeries—had neutralizing antibody titers of 128 and 512. These workers had unexplained febrile illnesses in the weeks after exposure to potentially infective material. Further details are provided in the article by Chant et al. in this issue.

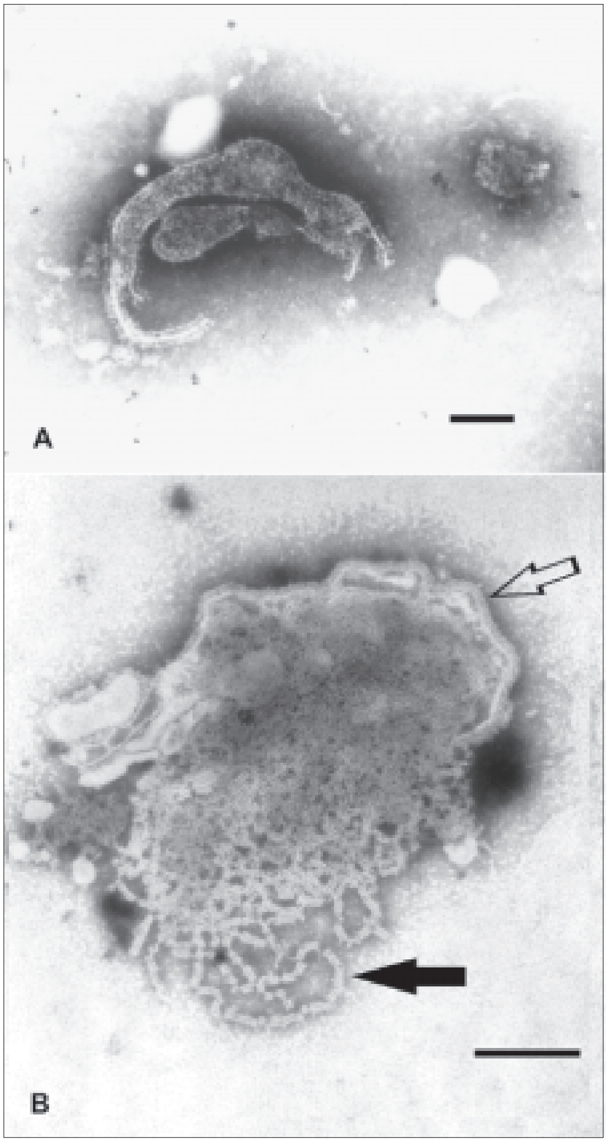

Figure. Transmission electron micrographs of Menangle virus negatively stained with 2% phosphotungstic acid. A. Depicts the pleomorphic nature of the virion; bar=100nm. B. Shows a disrupted virion, the virus envelope with surface projections (hollow arrow) and nucleocapsids (solid arrow); bar=100nm.

A large breeding colony of gray-headed fruit bats (*Pteropus poliocephalus*), as well as little red fruit bats (*P. scapulatus*), roosts within 200 m of the affected piggery from October to April; therefore, fruit bats were investigated as a potential source of infection. In a preliminary study, 42 of 125 serum samples collected from fruit bats in New South Wales and Queensland were positive in the virus neutralization test, with titers of 16 to 256. Positive samples were from 26 of 79 gray-headed fruit bats, 11 of 20 black fruit bats (*P. alecto*), 4 of 10 spectacled fruit bats (*P. conspicillatus*), 0 of 15 little red fruit bats, and one unidentified species. This panel included positive samples collected in 1996 before the pigs were infected, as well as positive samples collected in November 1997 from a colony of gray-headed fruit bats 33 km from the piggery, supporting the hypothesis that fruit bats were the primary source of the virus. Other species in the vicinity of the affected piggery, including rodents (n = 19), birds (n = 13), cattle (n = 60), sheep (n = 70), cats (n = 25), and a dog, were seronegative.

Along with EMV (7-11) and a lyssavirus causing encephalitis (14,15), this is the third virus causing disease in humans or domesticated animals that appears to have emerged from fruit bats in Australia in recent years. Menangle virus is proposed as a common name for this agent, should further studies confirm that it is a newly recognized virus.

### Acknowledgments

We thank Bryan Eaton, Peter Hooper, Jacquie Kattenbelt, Brenda King, Chris Morrissey, Paul Selleck, and Harvey Westbury from the CSIRO Australian Animal Health Laboratory, Geelong, for their invaluable contributions with serology, histopathology, and PCR and Keith Hart and Andrew Glover, Moss Vale Rural Lands Protection Board, Camden, for collection of serum samples from rodents, birds, cattle, sheep, and cats at the affected piggery. Sera from humans were provided by the Public Health Unit, South Western Sydney Area Health Service, Liverpool, under the directorship of Kerry Chant. Peter Young and Hume Field from the Animal Research Institute, Department of Primary Industries, Yeerongpilly, Queensland, kindly provided serum samples from fruit bats. We appreciate contributions by technical staff in the virology section and regional veterinary laboratory at the Elizabeth Macarthur Agricultural Institute, Menangle, and at the University of Sydney, Camden, throughout this investigation.

Adrian Philbey is a veterinary research officer at Elizabeth Macarthur Agricultural Institute, New South Wales. His research interests include veterinary pathology with a special interest in virology, use of molecular techniques for pathogen identification in domestic animals, vaccine development for infectious diseases in domestic animals, and rabbit calicivirus.

### References

1. Osterhaus ADME, Vedder EJ. Identification of virus causing recent seal deaths. Nature 1988;335:20.
2. Osterhaus ADME, Groen J, De Vries P, UytdeHaag FGCM. Canine distemper virus in seals. Nature 1988;335:403-4.
3. Kennedy S, Smyth JA, McCullough SJ, Allan GM, McNeilly F, McQuaid S. Confirmation of cause of recent seal deaths. Nature 1988;335:404.
4. Kennedy S, Smyth JA, Cush PF, McCullough SJ, Allan GM, McQuaid S. Viral distemper now found in porpoises. Nature 1988;336:21.
5. Domingo M, Ferrer L, Pumarola M, Marco A, Plana J, Kennedy S, et al. Morbillivirus in dolphins. Nature 1990;348:21.
6. Roelke-Parker ME, Munson L, Packer C, Kock R, Cleaveland S, Carpenter M, et al. A canine distemper virus epidemic in Serengeti lions (*Panthera leo*). Nature 1996;379:441-5.
7. Murray K, Selleck P, Hooper P, Hyatt A, Gould A, Gleeson L, et al. A morbillivirus that caused fatal disease in horses and humans. Science 1995;268:94-7.
8. Allworth T, O'Sullivan J, Selvey L, Sheridan J. Equine morbillivirus in Queensland. Communicable Diseases Intelligence1995;19:575.
9. Young P. Possible reservoir host of equine morbillivirus identified. Communicable Diseases Intelligence 1996;20:262.
10. Young PL, Halpin K, Selleck PW, Field H, Gravel JL, Kelly MA, et al. Serologic evidence for the presence in *Pteropus* bats of a paramyxovirus related to equine morbillivirus. Emerg Infect Dis 1996;2:239-40.
11. Halpin K, Young P, Field H. Identification of likely natural hosts for equine morbillivirus. Communicable Diseases Intelligence 1996;20:476.
12. Stephan HA, Gay GM, Ramirez TC. Encephalomyelitis, reproductive failure and corneal opacity (blue eye) in pigs, associated with a paramyxovirus infection. Vet Rec 1988;122:6-10.
13. Moreno-López J, Correa-Girón P, Martínez A, Ericsson A. Characterization of a paramyxovirus isolated from the brain of a piglet in Mexico. Arch Virol 1986; 91:221-31.
14. Fraser GC, Hooper PT, Lunt RA, Gould AR, Gleeson LJ, Hyatt AD, et al. Encephalitis caused by a lyssavirus in fruit bats in Australia. Emerg Infect Dis 1996;2:327-31.
15. Allworth A, Murray K, Morgan J. A human case of encephalitis due to a lyssavirus recently identified in fruit bats. Communicable Diseases Intelligence 1996;20:504.

## References

[PDF_00215] Domingo M., Ferrer L., Pumarola M., Marco A., Plana J., Kennedy S., McAliskey M., Rima B. K. (1990). Morbillivirus in dolphins.. Nature.

[PDF_00245] Fraser G. C., Hooper P. T., Lunt R. A., Gould A. R., Gleeson L. J., Hyatt A. D., Russell G. M., Kattenbelt J. A. (1996). Encephalitis caused by a Lyssavirus in fruit bats in Australia.. Emerg Infect Dis.

[PDF_00212] Kennedy S., Smyth J. A., Cush P. F., McCullough S. J., Allan G. M., McQuaid S. (1988). Viral distemper now found in porpoises.. Nature.

[PDF_00209] Kennedy S., Smyth J. A., McCullough S. J., Allan G. M., McNeilly F., McQuaid S. (1988). Confirmation of cause of recent seal deaths.. Nature.

[PDF_00242] Moreno-López J., Correa-Girón P., Martinez A., Ericsson A. (1986). Characterization of a paramyxovirus isolated from the brain of a piglet in Mexico.. Arch Virol.

[PDF_00222] Murray K., Selleck P., Hooper P., Hyatt A., Gould A., Gleeson L., Westbury H., Hiley L., Selvey L., Rodwell B. (1995). A morbillivirus that caused fatal disease in horses and humans.. Science.

[PDF_00206] Osterhaus A. D., Groen J., De Vries P., UytdeHaag F. G., Klingeborn B., Zarnke R. (1988). Canine distemper virus in seals.. Nature.

[PDF_00204] Osterhaus A. D., Vedder E. J. (1988). Identification of virus causing recent seal deaths.. Nature.

[PDF_00218] Roelke-Parker M. E., Munson L., Packer C., Kock R., Cleaveland S., Carpenter M., O'Brien S. J., Pospischil A., Hofmann-Lehmann R., Lutz H. (1996). A canine distemper virus epidemic in Serengeti lions (Panthera leo).. Nature.

[PDF_00238] Stephan H. A., Gay G. M., Ramírez T. C. (1988). Encephalomyelitis, reproductive failure and corneal opacity (blue eye) in pigs, associated with a paramyxovirus infection.. Vet Rec.

[PDF_00231] Young P. L., Halpin K., Selleck P. W., Field H., Gravel J. L., Kelly M. A., Mackenzie J. S. (1996). Serologic evidence for the presence in Pteropus bats of a paramyxovirus related to equine morbillivirus.. Emerg Infect Dis.

